# Analysis of the Oxidative Stress Status in Nonspecific Vaginitis and Its Role in Vaginal Epithelial Cells Apoptosis

**DOI:** 10.1155/2015/795656

**Published:** 2015-10-19

**Authors:** Zhaojie Chen, Zhen Zhang, Haiyan Zhang, Beibei Xie

**Affiliations:** Department of Gynaecology, Linyi People's Hospital, Linyi, Shandong 276000, China

## Abstract

Nonspecific vaginitis (NSV), also named bacterial vaginosis, is one of the most common genital system diseases in women during their reproductive years. The specific pathogenic mechanism of NSV is not clear yet. Upon the balance alteration, large amount of reactive oxidant species (ROS) is generated and accumulated in the genital tract, and thus resulting in oxidative stress, which has been reported to be an important trigger of mitochondrial pathway cell apoptosis. In this study, the antioxidant secretion level and antioxidant enzyme activity in the vaginal discharge were evaluated to analyze the oxidative status in the vaginal tract of NSV patients. The effect of oxidative stress on the vaginal mucosa epithelial cell apoptosis was then studied. The role of oxidative stress on NSV development was uncovered; thus open new direction for the prevention and treatment of NSV by providing antiradical agents was revealed.

## 1. Introduction

Vaginitis represents the most common problem in gynecology clinics, causing considerable public health expenditures plus emotional and social distress [1]. Large number of women in their reproductive ages are diagnosed with nonspecific vaginitis (NSV). Their vaginal discharge gives out a fishy odor, which is intensified after intercourse and during menstruation. NSV is diagnosed on the basis of the presence of at least three out of the following four parameters: presence of thin grey homogeneous discharge, vaginal pH ≥ 4.7, release of fishy odor after adding 10% potassium hydroxide (KOH), and the presence of “clue cells” [2].

Normally, a healthy vaginal environment maintains a balance between the protective organisms (*Lactobacillus*) and other anaerobic and aerobic flora, with* Lactobacillus* as the majority organism. The adversely altered balance between protective organisms and potential pathogens in the microenvironment of genital tract results in gynecological disease, such as NSV [3, 4]. In healthy women, H_2_O_2_ is produced by lactobacilli and maintained a typical concentration in the vaginal fluid. This typical concentration of H_2_O_2_ is toxic to many microorganisms and thus provides an intrinsic protective mechanism in the vaginal compartment [5]. In NSV patients, the number of lactobacilli is significantly reduced and anaerobic and aerobic flora have overgrowth. The anaerobic bacteria produce aminopeptidases to generate amines which contribute to the signs and symptoms of NSV, such as elevated vaginal pH and production of discharge as well as malodor. Further, the excessive amount of bacteria adheres to epithelial cell surfaces which results in “clue cells.” In addition, enzymes and metabolic by-products of the abnormal bacterial flora significantly impede normal white blood cell response to infection and other lesions. Several studies have documented an association between NSV and premature rupture of membranes, preterm delivery, or postpartum sepsis [6–8].

Oxidant stress is associated with the generation of reactive oxidant species (ROS) which are ultimately responsible for damage to a wide variety of cellular components (enzymes, DNA, membrane lipids, etc.). Oxidative stress has been shown to be a major player in many gynecological diseases, such as fibroids, endometriosis, and postoperative adhesions [9–11]. Han et al. found that* T. vaginalis* was able to stimulate the production of proinflammatory cytokine and nitric oxide (NO) and the expression of iNOS [12]. Ray and Chakrabarti found that the lipid peroxidation and antioxidant potential was altered in human uterine tumors [13]. However, the role of antioxidants in the vaginal discharge on vaginitis development has not been reported.

Apoptosis is a special form of cell death which can be triggered by a variety of signals and pathophysiological conditions, including oxidative stress [14, 15]. It has been reported that* T. vaginalis* induced neutrophilic apoptosis by activation of caspase 3 and reduction of Mcl-1 expression via ROS [16, 17]. Other studies have shown that oxidative stress is an important trigger of mitochondrial pathway of cell apoptosis [18]. ROS induced apoptosis effect via activating Jun N-terminal kinases (JNKs) signaling pathway [19, 20]. JNKs belong to the superfamily of MAP-kinases which play a critical role in death receptor-initiated extrinsic as well as mitochondrial intrinsic apoptotic pathways [20]. JNKs activate apoptotic signaling by upregulating proapoptotic genes via the transactivation of specific transcription factors or by directly modulating the activities of mitochondrial pro- and antiapoptotic proteins through distinct phosphorylation events. Regarding NSV, mucosa epithelial cell death or whether it is induced by oxidative stress has not been reported.

This study was intending to investigate the mechanism of NSV by analyzing the oxidative stress status in the vaginal discharge and its role on vaginal mucosa epithelial cell apoptosis. Specifically, the antioxidant level (malondialdehyde (MDA) and hydrogen peroxide (H_2_O_2_)) and antioxidant enzymes activity (superoxide dismutase (SOD) and catalase (CAT)) in the vaginal discharge were firstly measured. The viability of vaginal mucosa epithelial cell was then evaluated and finally, the expression level of apoptosis related proteins was studied. The purpose of this research is to find out the mechanism of NSV development and provide new ways for NSV treatment by providing antiradical agents.

## 2. Materials and Methods

### 2.1. Patients and Sampling

This study was performed at the outpatient clinic of Linyi People's Hospital. Before this study, the approval of the Medical Ethical Committee of Linyi People's Hospital was obtained. A total of 100 female patients (aging 20 ~ 47, average age 33.2 ± 5.33) with symptoms suggestive for NSV were involved in this study. Meanwhile, a total of 30 healthy females aged 22 ~ 45 years (average age 34.6 ± 4.56 years) participated in the control group. Written informed consent was obtained by each patient before starting the study. Patients with recent antimicrobial treatment, pregnancy, total hysterectomy, history of hypersensitivity to Vitamin C, and AIDS or HIV positive tests were excluded from the study. The diagnosis of NSV was stated as presence of at least three out of the following characteristic symptoms: presence of thin grey homogeneous discharge, a vaginal pH ≥ 4.7, release of fishy odor after adding 10% potassium hydroxide (KOH), and the presence of “clue cells.” NSV patients were treated with 200 mg oral metronidazole vaginal effervescent tablets and 250 mg Vitamin C vaginal tablets every day for one week.

An unmoistened sterile speculum was inserted to expose cervix uteri before any other vaginal examination was performed. Samples were collected from the cervix uteri wall using cotton swabs before and after the one-week treatment. Samples were immersed in 1 mL of sterilized phosphate buffer (PBS) and stored at −80°C for further analysis.

To analyze the oxidative stress, collected vaginal discharge was washed with 1 mL PBS. The cotton swab was discarded and the PBS was centrifuged at 10000 rpm/min for 20 min. The supernatant was used to measure the level of malondialdehyde (MDA) and hydrogen peroxide (H_2_O_2_) and activities of superoxide dismutase (SOD) and catalase (CAT). On the other hand, mucosa epithelial cells were collected from the vaginal tract for the analysis of cell apoptosis and apoptosis related proteins.

### 2.2. Analysis of Oxidative Stress Status in Vaginal Discharge

To analyze the oxidative status of the vaginal discharge, the content of MDA and H_2_O_2_ as well as the activities of CAT and SOD were measured. Specifically, the MDA level was measured as described by Yoshioka et al. to estimate tissue lipid peroxidation [21]. H_2_O_2_ content was analyzed using a hydrogen peroxide assay kit (Nanjing Jiancheng, China) following the manufacture's instruction. A catalase assay kit (Nanjing Jiancheng, China) and a total superoxide dismutase (T-SOD) assay kit (Nanjing Jiancheng, China) were utilized to determine CAT and SOD activity, respectively. The assays were performed according to the manufacturer's protocol.

#### 2.2.1. MDA Level

Malondialdehyde (MDA) is a product generated during lipid peroxide degradation and can be reacted with thiobarbituric acid (TBA) to produce chemicals with red color. These red chemicals can be detected by fluorospectrophotometry under 553 nm using an excitation wavelength of 515 nm. MDA level was measured as described by Yoshioka et al. to estimate tissue lipid peroxidation (Yoshioka, 1979). Specifically, 50 *μ*L of the supernatant was mixed with 4.0 mL H_2_SO_4_ (0.04 M/L) and 500 *μ*L phosphotungstic acid (10% w/v). After 5 min, the mixture was centrifuged and supernatant was discarded. The obtained product was then mixed with 1.0 mL D.I. water and was vibrated for 1 min. 1.0 mL TBA (0.67% w/v) was then added to the mixture and incubated at 100°C for 1 h. After cooling down to room temperature, 4.0 mL water saturated N-butyl alcohol was added to the solution and vibrated for 1 min. The mixture was then centrifuged for 5 min at 2000 rpm. 3 mL of the supernatant was used to measure the fluorescence intensity with 515 nm and 553 nm as excitation and emission wavelength, respectively. The concentration of MDA was calculated as

(1) MDA concentration=21×Fluorescence intensity of sampleFluorescence intensity of standard.

#### 2.2.2. H_2_O_2_ Content

Hydrogen peroxide (H_2_O_2_) can be reacted with chromogenic agent to produce a complex which can be detected using spectrometer at 405 nm. The concentration of H_2_O_2_ can be calculated from the absorbance. In this study, H_2_O_2_ content was analyzed using a hydrogen peroxide assay kit (Nanjing Jiancheng, China) following the manufacture's instruction.

#### 2.2.3. CAT Activity

The decomposition of hydrogen peroxide (H_2_O_2_) catalyzed by catalase (CAT) can be quickly suspended by ammonium molybdate. The unreacted amount of H_2_O_2_ reacted with ammonium molybdate to produce a pale yellow complex which can be detected using a spectrophotometry at 405 nm. In our study, CAT activity was measured using a catalase assay kit (Nanjing Jiancheng, China) following the manufacturer's protocol.

#### 2.2.4. SOD Activity

Hydroxylamine can be autooxidized to produce super oxygen anion under aerobic conditions (O_2_^−^), and these oxygen anions can be detected by NBT. In the presence of SOD, the O_2_^−^ will decompose and NBT reduction is suppressed. In this way, the SOD activity can be determined by measuring the amount of NBT reduction product using colorimetric method at 560 nm. In this study, a total superoxide dismutase (T-SOD) assay kit (Nanjing Jiancheng, China) was utilized to determine SOD activity in the supernatant and experiment was performed following the manufacturer's protocol.

### 2.3. Cell Viability and Apoptosis of Mucosa Epithelial Cells

A lactate dehydrogenase assay kit (Nanjing Jiancheng, China) was used to study the lactate dehydrogenase (LDH) activity in the mucosa epithelial cells, while the cell apoptosis was evaluated by DNA ladder experiment. Specifically, obtained mucosa epithelial cells were washed with 0.01 M PBS (pH 7.4) for three times before lysis in ladder cell lysis solution for 1 h at 50°C. RNase A (0.25 mg/mL) was then added to the mixture and incubated for 1 h at 50°C. The mixture was centrifuged at 13000 rpm for 10 min. The supernatant was then mixed with twice of ethanol in volume and placed at −80°C overnight. It was then centrifuged at 13000 rpm for 20 min and the supernatant was discarded. The obtained product was dry naturally. They were then resuspended in 20 *μ*L TE buffer for agar gel electrophoresis.

### 2.4. Detection of Apoptosis Related Proteins

Western blot analysis was used to identify the activated caspase 3 and apoptosis related mitochondrial pathway proteins, including cytochrome C, Bcl-2, and Bax. Specifically, mucosa epithelial cells were lysed in Laemmli buffer. Equal amounts of protein samples (10 mg) were separated by SDS-PAGE and transferred onto nitrocellulose membranes. The primary antibodies for caspase 3, cytochrome C, Bcl-2, and Bax were purchased from BD Biosciences. All other antibodies were from Cell Signaling. After incubation with peroxidase-linked/horseradish peroxidase-labeled secondary antibodies, specific bands were visualized using the ECL enhanced chemiluminescence Western blotting detection reagents (Nanjing Jiancheng, China). The optical density of the corresponding Western blot band was measured using Kodak ID Image software. Western blot analysis of *β*-actin was also performed.

### 2.5. Statistical Analysis

The statistical analysis has been performed by using *χ* ^2^ test and Fisher exact test, Wilcoxon test, or Student's *t*-tests, according to the data distribution.

## 3. Results

### 3.1. Symptoms and Signs of Vaginal Discharge

Table 1 shows the demographic data of vaginal discharge from NSV patients and controls. It can be seen that, in all of the vaginal discharge of the 100 NSV cases, more than 97% patients showed all of the four NSV characteristic symptoms, including presence of thin grey homogeneous discharge, a vaginal pH ≥ 4.7, release of fishy odor after adding 10% potassium hydroxide (KOH), and the presence of “clue cells.” However, these symptoms were seldom observed in healthy women. Regarding the NSV patients, the presence of characteristic symptoms was greatly decreased to a level similar to that of healthy women after the one-week treatment with metronidazole vaginal effervescent tablets and Vitamin C vaginal tablets.

### 3.2. Analysis of Oxidative Stress Status in Vaginal Discharge

MDA and H_2_O_2_ content and CAT and SOD activities were measured to analyze the oxidative stress status in the vaginal discharge (Table 2). The MDA level in NSV patients was 35.37 ± 19.14 mM/L which is almost three times the control. Since MDA is a major product of lipid peroxide degradation, the significantly higher concentration of MDA indicates the large amount of cell membrane in the vagina of NSV patients. The H_2_O_2_ level in the vaginal discharge collected from NSV patients before any treatment (23.26 ± 17.32 mM/L) was almost 10 times higher than those from healthy women (2.89 ± 2.47 mM/L), suggesting a serious oxidative stress environment has been established in the vagina. After treating with antibacterial (metronidazole) and antioxidant (Vitamin C) agent, the patients' vaginal MDA and H_2_O_2_ level was effectively decreased to a level close to that of control.

From Table 2, the CAT activity of vaginal discharge from NSV patients was around 1.66 ± 0.82 U/mL, which is significantly lower than that of healthy women (5.03 ± 3.88 U/mL) (*P* < 0.01). For the SOD activity, NSV patients showed a value (21.44 ± 18.02 U/mg) more than twice smaller than that of healthy women. However, after the one-week drug treatment, both CAT and SOD activities were recovered to the levels similar to control.

### 3.3. Cell Viability and Apoptosis of Mucosa Epithelial Cells

**Figure 1:**
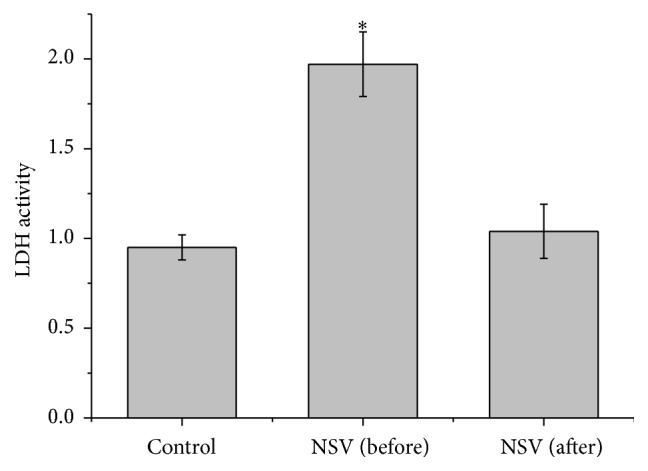
LDH activity of mucosa epithelial cells collected from the vagina of healthy women and patients with NSV before and after treatment with metronidazole vaginal effervescent tablets and Vitamin C vaginal tablets. Data was presented as folds change relative control.  ^*∗*^Significantly different from control, *P* < 0.05.

It was reported that the mucosa epithelial cells in NSV undergo apoptotic cell death [19]. In order to find out the role of NSV in mucosa epithelial cell apoptosis, which cell viability was accessed by LDH activity. From Figure 1, it can be seen that the LDH activity of mucosa epithelial cells in NSV patients was significantly higher than that of healthy women (Figure 1). Since LDH activity in the cell medium was frequently used as an indicator for necrosis, high LDH activity is correlated with large amount of cell death. In this study, the significantly higher LDH activity in NSV patients in this study suggested that more mucosa epithelial cells in NSV group underwent cell death, indicating that NSV was highly possible major reason responsible for mucosa epithelial cell death.

**Figure 2:**
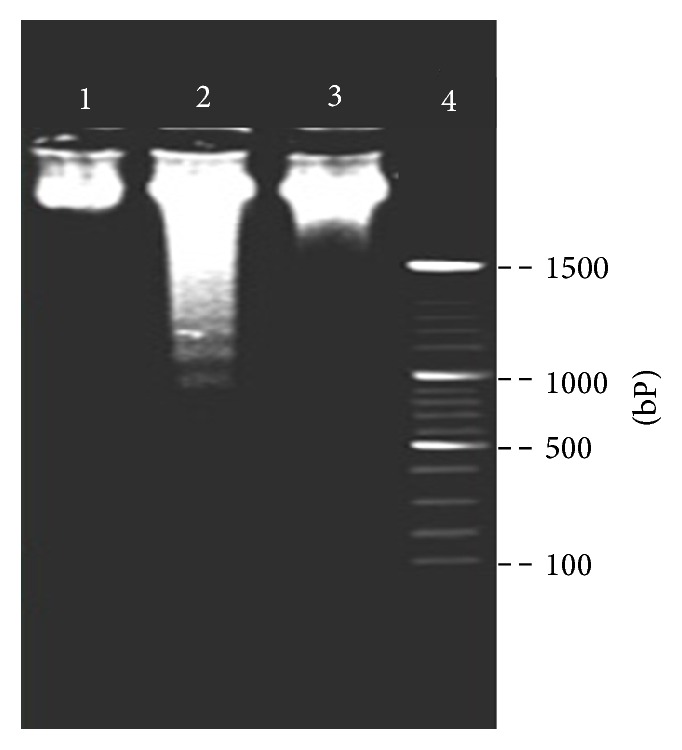
DNA ladder results of mucosa epithelial cells collected from the vagina of healthy women and patients with NSV before and after treatment with metronidazole vaginal effervescent tablets and Vitamin C vaginal tablets. (1) Control, (2) NSV (before), (3) NSV (after), and (4) marker.

Destruction of nuclear and damage or breakage of DNA resulted in the formation of DNA ladder, which is an indicator of cell apoptosis. In order to find out whether mucosa epithelial cells undergo apoptosis, DNA ladder experiment was carried out in this study. From Figure 2, obvious DNA ladder was observed in mucosa epithelial cells from NSV patients, but not in cells from healthy women. The result suggested that mucosa epithelial cells in NSV patient possibly undergo an apoptosis cell death way.

### 3.4. Expression of Apoptosis Related Proteins

**Figure 3:**
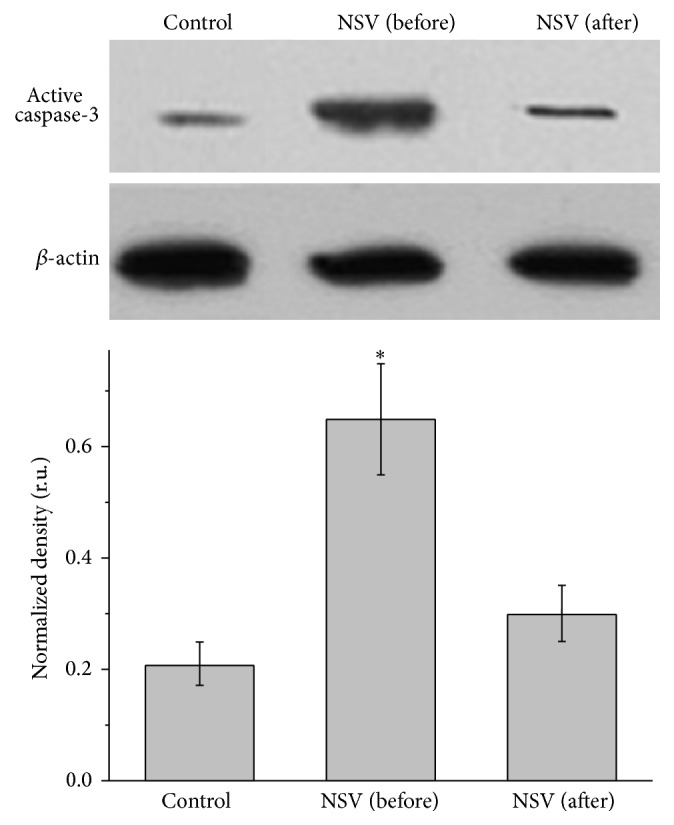
Western blot analysis of active caspase 3 in mucosa epithelial cells collected from the vagina of healthy women and patients with NSV before and after treatment with metronidazole vaginal effervescent tablets and Vitamin C vaginal tablets followed by statistical analysis of the optical band density relative to *β*-actin (relative units).  ^*∗*^Significantly different from control, *P* < 0.05.

Expression of active caspase 3 and apoptosis related mitochondrial pathway proteins, including Bcl-2, Bax, and cytochrome C, was analyzed using Western blotting. As it was shown in Figure 3, the amount of active caspase 3 was significantly higher in NSV patients (0.68 in band density relative to *β*-actin) comparing to control (0.21 in band density relative to *β*-actin) (*P* < 0.05). Since activation of caspase proteins, especially caspase 3, is an important indicator of cell apoptosis, these results suggested that apoptosis of mucosa epithelial cells happened in the vagina of NSV patients. However, after treating with metronidazole vaginal effervescent tablets and Vitamin C vaginal tablets, the expression level of active caspase 3 in NSV patients decreased to a level (0.28 in band density relative to *β*-actin) close to that of control (0.21 in band density relative to *β*-actin).

**Figure 4:**
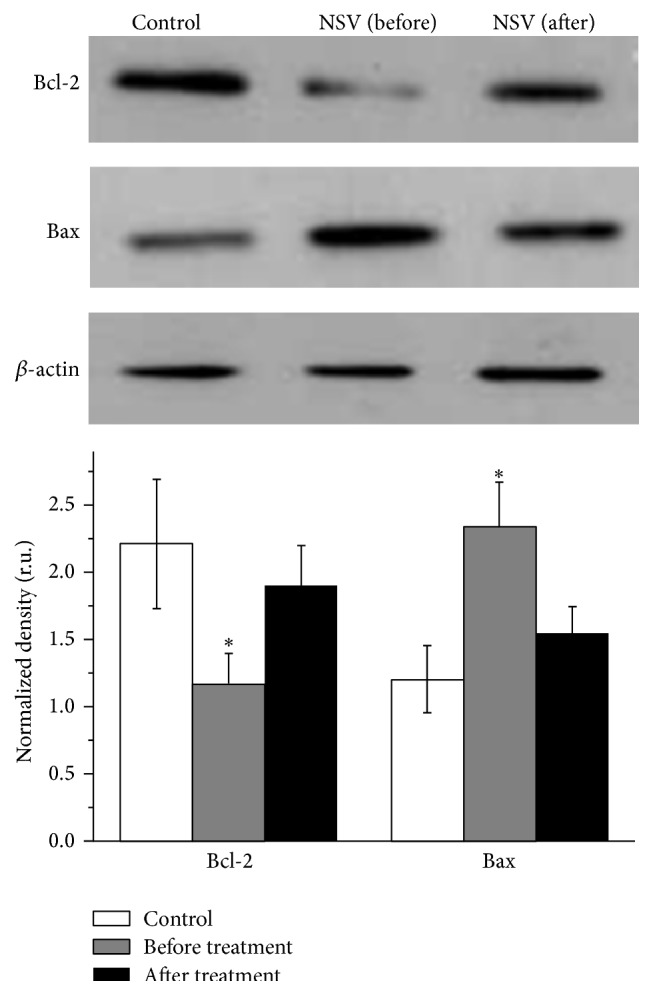
Western blot analysis of apoptosis related proteins Bcl-2 and Bax in mucosa epithelial cells collected from the vagina of healthy women and patients with NSV before and after treatment with metronidazole vaginal effervescent tablets and Vitamin C vaginal tablets followed by statistical analysis of the optical band density relative to *β*-actin (relative units).  ^*∗*^Significantly different from control, *P* < 0.05.

Comparing to healthy women, the expression of Bcl-2 in mucosa epithelial cells from NSV patients was significantly decreased (*P* < 0.05) (Figure 4). Regarding Bax, more than twice the elevation in protein expression has been found in NSV patients compared to healthy women. However, the changes in protein expression of Bcl-2 and Bax could be reversed by metronidazole vaginal effervescent tablets and Vitamin C vaginal tablets treatment.

**Figure 5:**
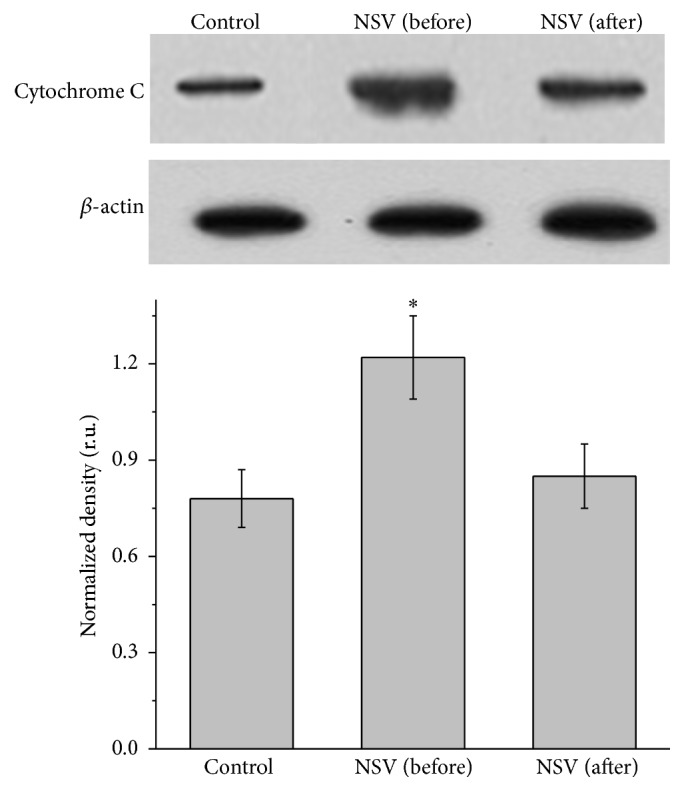
Western blot analysis of cytochrome C in mucosa epithelial cells collected from the vagina of healthy women and patients with NSV before and after treatment with metronidazole vaginal effervescent tablets and Vitamin C vaginal tablets followed by statistical analysis of the optical band density relative to *β*-actin (relative units).  ^*∗*^Significantly different from control, *P* < 0.05.

Regarding cytochrome C, it was significantly increased to a level of 1.22 relative units in band density when women with NSV were detected, which was much higher than that of healthy women (0.79 relative units) (Figure 5). The elevated cytochrome C released indicated that the membrane of mitochondrial was changed to allow the release of cytochrome C. Similar to Bax and Bcl-2, this change of cytochrome C expression can be recovered to a normal level by treating with drugs. Bcl-2 proteins are important regulators of the membrane permeability of mitochondria which plays an important role in the release of cytochrome C. The low level of Bcl-2 detected in our study allowed more cytochrome C release from mitochondria, and elevated level of cytochrome C has been observed.

## 4. Discussion

NSV, assessed as presence of 3 symptoms out of discharge, malodor, vaginal pH > 4.7, and “clue cells,” has brought many troubles to the patients' everyday life. Normally, lactobacilli produce a small amount of H_2_O_2_ which is toxic to many microorganisms and thus provides an intrinsic protective mechanism in the vaginal compartment. MDA, as a free radical, is able to modify biomolecular and induce oxidative damage to microorganisms, thus beneficial to the protective effect of H_2_O_2_. However, if the highly deleterious H_2_O_2_ is overproduced, it will cause oxidation of cellular targets such as DNA, proteins, and lipids leading to mutagenesis and cell death [5]. In this study, it was found that the concentration of MDA and H_2_O_2_ in the virginal discharge from NSV patients was significantly higher than that from healthy women, which is possible because the overgrowth of pathogens induces oxidative stress in the vaginal tract of NSV patients. Under oxidative stress, large amount of ROS is produced. The excessive ROS oxidized the cellular molecules and generated large amount of H_2_O_2_ and MDA. The overproduction of H_2_O_2_ resulted in oxidative damage to mucosa epithelial cells and even cell death. On the other hand, the excess MDA created damage to normal epithelial cells and further induced ROS production [22]. Additionally, in the vaginal tract, ROS attacks the vagina mucosa and induces the mucosa epithelial cell death.

Several antioxidant enzymes, for example, SOD and CAT, have been shown to be protective against oxidative stress [23]. SOD catalyzes the dismutation reaction of superoxide ion (O_2_^•−^) to H_2_O_2_. CAT scavenges the toxic H_2_O_2_ by catalyzing its decomposition into O_2_ and H_2_O. Thus high CAT activity is critical to ensure the SOD function [24]. Comparing to healthy women, significantly lower SOD activity and lower CAT activity have been observed from NSV patients and these alterations were able to recover via metronidazole and Vitamin C treatment (Table 2). All these results suggest that oxidative stress was created in the vaginal microenvironment of NSV patients and affected the NSV development.

ROS generated under oxidative stress have been implicated in the activation of various signaling molecules and induce dysfunction of mitochondria and other organelles [25, 26]. We speculated that oxidative stress in vaginal tract might result in apoptosis of mucosa epithelial cells, and thus the cell viability and DNA ladder assay were performed in this study. It was found that the viability of epithelial cells from NSV patients was significantly lower than that of healthy women and obvious DNA ladder has been observed (Figures 1 and 2), implicating that apoptosis cell death has been triggered on mucosa epithelial cells in NSV patients.

The activation of cell apoptosis is directly related to the activation of caspase family; thus the activation of caspase proteins, especially caspase 3, has been widely used as the indicator of cell apoptosis. Large amount of cleaved caspase 3 usually indicates the occurrence of cell apoptosis. From Figure 3, the amount of active caspase 3 in mucosa epithelial cells from NSV patients was significantly larger than control and after drug treatment, indicating that apoptosis happened on mucosa epithelial cells in NSV patients.

Previous reports emphasized the importance of mitochondria in ROS induced apoptosis of various cell types [27]. When cells were stimulated by apoptosis signals, the membrane permeability of mitochondria was changed, resulting in the release of cytochrome C into cytoplasm [27]. This cytochrome C activates caspase families which induce cell apoptosis. Bcl-2 family proteins are the most important regulator of the membrane permeability of mitochondria [28]. Among them, Bcl-2 is normally anchored on the membrane of mitochondria to inhibit the release of cytochrome C. When cells were stimulated by apoptosis signals, Bcl-2 was released from the membrane to allow the release of cytochrome C into cytoplasm. Bcl-2 and cytochrome C together induce the mitochondria pathway triggered apoptosis. Bax has been shown to homodimerize or heterodimerize with Bcl-2. Excess Bax counters the ability of Bcl-2 to repress cell death [29]. In this study, it was found that the concentration of Bcl-2 in mucosa epithelial cells of NSV patients is greatly reduced indicating that the inhibition of cytochrome C release became weaker. The detected Bax was significantly elevated and thus further confirmed ability of Bcl-2 to repress cell death was decreased. Finally, high cytochrome C level detected in our study shows that mitochondria pathway apoptosis of mucosa epithelial cell death happened in NSV patients' vaginal tract. Finally, the decrease of Bcl-2 and increase of Bax and cytochrome C were controllable via the metronidazole and Vitamin C treatment. It is possible that antioxidant, for example, Vitamin C, might work via releasing the oxidative stress formed in NSV patients and increasing patients' immunity against the pathogen. In this way, the curative effect of metronidazole is strengthened.

## 5. Conclusion

In this study, high concentration of MDA and H_2_O_2_, elevated SOD activity, and reduced CAT activity were observed in the vaginal discharge from NSV patients, indicating that oxidative stress was formed in their vaginal microenvironment. Further, the decreased cell viability, obvious DNA ladder, and increased active caspase 3 content suggested that the vaginal mucosa epithelial cells in NSV patients undergo apoptosis. Further study on the concentration of Bcl-2, Bax, and cytochrome C confirmed that mitochondrial pathway induced apoptosis was triggered on the vaginal mucosa epithelial cells in NSV patients. We also found that the oxidative stress induced cell apoptosis is able to be controlled by adding antioxidant, for example, Vitamin C, into conventional metronidazole treatment.

## Figures and Tables

**Table 1:** Demographic data and symptoms and signs of vaginal discharge from 100 NSV patients and 30 healthy women involved in this study.

Variable	NSV		Control
	Before treatment	After treatment	
Age (years)	33.2 ± 5.33		34.6 ± 4.56
Discharge (%)	100	16	4
Malodor (%)	97	6	0
pH ≥ 4.7 (%)	100	12	2
Clue cells (%)	100	13	0

**Table 2:** MDA and H_2_O_2_ contents and CAT and SOD activities of vaginal discharges collected from the vagina of healthy women and patients with nonspecific vaginitis before and after treatment with metronidazole vaginal effervescent tablets and Vitamin C vaginal tablets.

Variable	NSV		Control
	Before treatment	After treatment	
MDA (mM/L)	35.37 ± 19.14	10.14 ± 7.65	9.87 ± 5.96
H_2_O_2_ (mM/L)	23.26 ± 17.32	2.89 ± 2.47	2.54 ± 2.15
CAT (U/mL)	1.66 ± 0.82	4.33 ± 3.02	5.03 ± 3.88
SOD (U/mg)	21.44 ± 18.02	45.58 ± 29.64	58.60 ± 30.32
